# Effects of a Single Intramuscular Dose of Rasburicase on Plasma Uric Acid Concentrations in Fasted and Fed Ball Pythons (*Python regius*)

**DOI:** 10.3390/vetsci13070645

**Published:** 2026-06-30

**Authors:** Andrés Montesinos Barceló, Alicia Tortosa García, María Ardiaca García

**Affiliations:** 1 Exotic Animals Veterinary Hospital Medivet-Los Sauces, Santa Engracia 63, 28010 Madrid, Spain; ardiaca.m@outlook.com; 2 Complutense Veterinary Teaching Hospital, Complutense University of Madrid, Avda. Puerta De Hierro s/n, 28040 Madrid, Spain; alictort@ucm.es

**Keywords:** ball python, *Python regius*, hyperuricemia, uric acid, rasburicase, gout, reptile medicine, renal disease, urate metabolism

## Abstract

**Background:** Hyperuricemia is a common problem in captive reptiles and is associated with gout and renal disease. Rasburicase is a recombinant urate oxidase that converts uric acid into allantoin, a more water-soluble metabolite. This study evaluated the safety and efficacy of rasburicase in clinically healthy ball pythons (*Python regius*). **Methods**: Eight adult ball pythons were included in a prospective crossover study. Six experimental conditions were evaluated: fasting with and without rasburicase (0.2 mg/kg IM), feeding with a day-old chick with and without rasburicase, and feeding with a mouse with and without rasburicase. Plasma uric acid concentrations were measured at 0, 24, 48, 72, and 96 h. **Results**: Rasburicase did not significantly affect fasting uric acid concentrations. Feeding increased plasma uric acid concentrations, with peak values occurring between 24 and 72 h. Following chick consumption, rasburicase promoted a faster decline in UA concentrations, although differences were not significant (*p* = 0.907). Following mouse consumption, rasburicase significantly reduced UA concentrations (*p* = 0.002), with a mean reduction of 37.85%. No changes were observed in behavior, appetite or defecation. **Conclusions**: Rasburicase was well tolerated and reduced postprandial uric acid concentrations in ball pythons.

## Simple Summary

Hyperuricemia, defined as an abnormal increase in blood uric acid concentrations, is a frequently encountered disorder in captive reptiles and can lead to gout and kidney disease. Treatment options are limited, and some currently available drugs have uncertain efficacy or may be associated with adverse effects. Rasburicase is an enzyme that rapidly converts uric acid into allantoin, a more water-soluble compound that can be eliminated more easily by the body. However, scientific information on its efficacy and safety in reptiles remains scarce. This study evaluated the safety and uric acid-lowering effect of a single intramuscular dose of rasburicase (0.2 mg/kg) in eight clinically healthy ball pythons (*Python regius*). Plasma uric acid concentrations were measured during fasting and after feeding with either a thawed day-old chick or a mouse, both with and without rasburicase treatment. Rasburicase produced no measurable effect on baseline plasma uric acid concentrations in fasting snakes. However, after feeding, particularly after thawed mouse consumption, rasburicase significantly reduced plasma uric acid concentrations and accelerated their return to baseline values. No adverse effects or changes in behavior, appetite, or defecation were observed during the study. These findings suggest that rasburicase is safe and may be a useful therapeutic option for reducing increased uric acid concentrations in ball pythons. Further studies are needed to investigate its pharmacokinetics, long-term safety, and clinical efficacy in reptiles affected by hyperuricemia or gout.

## 1. Introduction

Renal disease is one of the most common medical conditions encountered in captive reptiles, both in pet animals and zoological collections [1,2,3]. It is also among the most frequently reported post-mortem findings in captive reptiles, with prevalence figures reaching 11.4% in some zoological collections [1]. Numerous factors may contribute to the development of renal disease, and its etiology is often multifactorial. Unfortunately, clinical signs are usually nonspecific and may remain absent until the disease has progressed to a life-threatening stage [4].

Renal disease in reptiles is associated with nonspecific clinical signs, including anorexia, weight loss, dehydration, and lethargy. In uricotelic species, plasma uric acid (UA) concentration is one of the main parameters used to assess renal function. However, plasma UA concentration does not directly reflect glomerular filtration rate, but rather tubular excretory capacity. In some reptile species, urea and ammonia concentrations may also contribute to the evaluation of renal function [4]. In addition, studies have validated the use of symmetric dimethylarginine (SDMA) concentrations to assess glomerular filtration rate in some reptile species [3]. Nevertheless, postprandial increases in plasma UA concentrations may occur in carnivorous species. In healthy reptiles, plasma UA concentrations may rise to levels approaching those observed in animals with renal disease and/or gout. In human medicine, UA concentrations above 6 mg/dL are considered a risk factor for gout, and gout has been reported in snakes with plasma UA concentrations of 28 mg/dL; however, postprandial plasma UA concentrations in snakes may reach values as high as 21 mg/dL [5].

In blood, UA is predominantly present as monosodium urate. An increase in UA concentrations due to overproduction or impaired elimination is referred to as hyperuricemia. Hyperuricemia may lead to disorders such as gout, in which monosodium urate precipitates and forms crystals within tissues. In addition, dehydration may promote precipitation of UA within the renal tubules, potentially resulting in tubular damage and destruction, renal injury, and ultimately renal failure. Furthermore the solubility of monosodium urate is pH- and temperature-dependent, which is clinically relevant in ectotherms whose body temperature varies substantially [5].

Hyperuricemia may result from chronic kidney disease or, less commonly, dehydration. Inadequate husbandry conditions, including insufficient humidity and inappropriate environmental temperatures, may contribute to the development of both conditions. In addition, the increased lifespan of reptiles maintained in captivity may contribute to a higher prevalence of chronic kidney disease in aged animals [1]. Treatment of hyperuricemia has traditionally relied on allopurinol to reduce UA production and on probenecid to enhance urinary UA excretion [2,3,4].

Allopurinol is an enzyme inhibitor that specifically inhibits xanthine oxidase, the enzyme responsible for catalysing the conversion of hypoxanthine to xanthine and xanthine to UA. Consequently, it is used to reduce UA production [4,5,6,7]. The principal metabolite of allopurinol is oxypurinol, which has a longer plasma half-life and is responsible for most of its therapeutic activity. Excretion occurs primarily through the kidneys; therefore, oxypurinol may accumulate in animals with impaired renal function [8]. Xanthine accumulation secondary to allopurinol therapy has been implicated in renal injury in carnivorous birds [9]. In green iguanas (*Iguana iguana*), oral administration of allopurinol at 20 mg/kg for 7 days has been reported to reduce UA concentrations by 41–45% [10]. In Hermann’s tortoises (*Testudo hermanni*), Russian tortoises (*Agrionemys horsfieldii*), and Greek tortoises (*Testudo graeca*), oral administration of allopurinol at 50 mg/kg every 72 h for three years reportedly reduced UA concentrations and promoted reduction or complete resolution of renal tophi [11].

Probenecid decreases UA reabsorption at the renal tubular level, thereby increasing UA excretion. Consequently, urate deposits are reduced, and dissolution of existing deposits is promoted. In humans, UA is filtered through the glomerulus, subsequently reabsorbed in the renal tubules, and then actively secreted again. However, there is no evidence that UA reabsorption occurs along the reptilian nephron [12]. Therefore, the use of probenecid in reptiles is considered contraindicated, and its efficacy in these species remains questionable. An anecdotal report described the administration of allopurinol at 20 mg/kg in combination with intracoelomic lactated Ringer’s solution and probenecid at 250 mg/kg in Greek tortoises (*Testudo graeca*), resulting in decreased plasma UA concentrations 45 days after completion of therapy. However, the observed efficacy was arguably attributable primarily to allopurinol rather than to probenecid [13].

Colchicine is a plant-derived alkaloid obtained from species of the genus Colchicum, commonly known as Autumn crocus. In human medicine, it is used for the treatment of acute gout and for the prevention of gout flares [14]. Colchicine binds to α- and β-tubulin, thereby inhibiting microtubule formation and modulating several inflammatory pathways involved in gout. It also interferes with activation of the NLRP3 inflammasome induced by monosodium urate (MSU) crystals, thereby reducing the release of active IL-1β, a key mediator of pain and inflammation in gout. In addition, colchicine affects neutrophil function by reducing adhesion and migration to inflammatory sites, and inhibits mast cell degranulation, thereby limiting the release of inflammatory mediators. Beyond suppressing pro-inflammatory pathways, colchicine may also promote anti-inflammatory mechanisms [14]. Pharmacokinetic data for colchicine in reptiles are currently lacking. Empirical use of colchicine in reptiles with gout has been described as an analgesic alternative when renal function is compromised and the use of nonsteroidal anti-inflammatory drugs (NSAIDs) is contraindicated [4].

Rasburicase is a recombinant enzyme produced from a genetically modified strain of *Saccharomyces cerevisiae* [15]. This enzyme catalyses the oxidative conversion of UA into allantoin, a highly water-soluble compound that is readily eliminated through the kidneys. Hydrogen peroxide is generated as a by-product of this reaction. When hydrogen peroxide concentrations exceed physiological levels, its neutralization depends on endogenous antioxidant mechanisms [16]. The pharmacokinetics of rasburicase have been studied primarily in children and young adults, who represent the main population receiving this treatment. In humans, rasburicase has a half-life of approximately 19 h and a volume of distribution like blood volume. It is metabolized through peptide hydrolysis, and its clearance is independent of renal function, because this interspecies extrapolation is uncertain. [17].

In human medicine, rasburicase is primarily used to treat hyperuricemia associated with tumor lysis syndrome (TLS) [15,17,18]. Gout is mostly treated with allopurinol; however, alternative therapies may be required in cases of allergy or drug incompatibility. In such situations, rasburicase has been used because of its ability to reduce tophus volume [17]. In hyperuricemic human patients, normalization of UA concentrations occurs within approximately 4 h following rasburicase administration, compared with approximately 24 h following allopurinol treatment [19]. Rasburicase has been associated with adverse effects in human patients, including severe complications such as haemolytic anemia, haemolysis, and methemoglobinemia. Individuals with glucose-6-phosphate dehydrogenase (G6PD) deficiency, an enzyme essential for protecting erythrocytes against oxidative stress, are particularly predisposed to these adverse reactions [20].

Few clinical studies evaluating the use of rasburicase in reptiles are currently available. One study conducted in red-eared sliders (*Trachemys scripta*) and Greek tortoises (*Testudo graeca*) reported reductions in UA concentrations of up to 70% within the first 24 h, with effects persisting for approximately 21 days [21]. In addition, the use of rasburicase has been described in a bearded dragon (*Pogona vitticeps*), in which daily intramuscular administration for five consecutive days reduced UA concentrations from the first day of treatment, with values remaining within normal limits for up to 25 days thereafter [22].

To date, studies evaluating the use of rasburicase in reptiles have been published only as conference abstracts and not in peer-reviewed journals. Therefore, the present study was designed to investigate the use of rasburicase in this taxonomic group. Ball pythons (*Python regius*) represent a suitable model for evaluating the potential effects of rasburicase in reptiles because they are carnivorous and uricotelic, and they exhibit marked postprandial variation in plasma uric acid concentrations.

The aim of the present study was to assess the safety and efficacy of intramuscular administration of rasburicase at a dose of 0.2 mg/kg for reducing plasma uric acid concentrations in clinically healthy ball pythons. We hypothesized that rasburicase administration would decrease plasma uric acid concentrations under both fasting and postprandial conditions. In addition, this study sought to evaluate the potential of rasburicase as a therapeutic option for the management of hyperuricemia and associated disorders, such as gout, in this species, for which current treatment options have important practical limitations.

## 2. Materials and Methods

### 2.1. Study Animals and Husbandry

Eight adult ball pythons (*Python regius*) were included in this study. The animals had a mean body weight of 1055 ± 230 g and comprised six males and two females, all aged between 7 and 8 years. The snakes were housed individually in separate terrariums and provided with water ad libitum. Each enclosure was equipped with a hide box and absorbent paper substrate. Environmental conditions included a 12 h light:12 h dark photoperiod, a source of ultraviolet light (Solar Raptor HDI 35 W, Cologne, germany, ECONLUX GmbH, Cologne, Germany, 272 µW/cm^2^), and a temperature gradient ranging from 28 to 33 °C. The maintenance diet consisted of thawed day-old chicks (*Gallus gallus domesticus*) offered every two weeks. The animals were privately owned and informed owners’ consent was obtained. Body weight was monitored throughout the study period to confirm that the snakes maintained stable body weight.

### 2.2. Therapeutic Trial

The study comprised six experimental conditions. Two conditions were established in fasted animals, whereas four were established after feeding. The order of the experimental conditions was fixed. Although the sequence may have influenced the results, the order described below was determined by the availability of the animals, which belonged to a privately owned collection. A minimum washout period of two weeks was maintained between consecutive experimental conditions. Each experimental condition included eight snakes, with five serial measurements obtained from each animal. The six protocols were as follows:

1. **Fasting without rasburicase (FNR):** At the end of a 2-week fasting period, the first blood sample was collected and designated as time 0. Additional blood samples were collected at 24, 48, 72, and 96 h. After the 96-h sample, the snakes were fed according to their normal husbandry routine.
2. **Fasting with rasburicase (FR):** This protocol was conducted at the end of a 2-week fasting period and after a 15-day washout period following the previous experimental condition. Blood was collected at time 0, immediately followed by intramuscular administration of rasburicase at 0.2 mg/kg using a 1.5 mg/mL formulation (Fasturtec^®^, Sanofi Winthrop Inc., Angni, Italy). The selected dose, 0.2 mg/kg administered intramuscularly, corresponds to the dose used in human pediatrics and that previously used in studies in reptiles [21,22]. Additional blood samples were collected at 24, 48, 72, and 96 h. After the 96-h sample, the snakes were fed according to their normal husbandry routine.
3. **Chick without rasburicase (CNR):** This protocol was conducted after a 2-week washout period following the previous experimental condition. Each snake was fed one day-old chick (mean body mass 35 g) 24 h before collection of the first blood sample. Therefore, the time 0 sample was obtained 24 h after chick ingestion. Additional blood samples were collected at 24, 48, 72, and 96 h.
4. **Chick with rasburicase (CR):** This protocol was conducted after a 2-week washout period. Each snake was fed one day-old chick 24 h (mean body mass 35 g) before collection of the first blood sample. Blood collection at time 0 was immediately followed by intramuscular administration of rasburicase at 0.2 mg/kg using a 1.5 mg/mL formulation (Fasturtec^®^, Sanofi Winthrop Inc.). Additional blood samples were collected at 24, 48, 72, and 96 h.
5. **Mouse without rasburicase (MNR):** This protocol was conducted after an additional 2-week washout period. Each snake was fed a thawed mouse (mean body mass 40 g) 24 h before collecting the first blood sample. Therefore, the time 0 sample was obtained 24 h after prey ingestion. Additional blood samples were collected at 24, 48, 72, and 96 h.
6. **Mouse with rasburicase (MR):** This protocol was conducted after a 2-week washout period. Each snake was fed a thawed mouse (mean body mass 40 g) 24 h before collecting the first blood sample. Blood collection at time 0 was immediately followed by intramuscular administration of rasburicase at 0.2 mg/kg using a 1.5 mg/mL formulation (Fasturtec^®^, Sanofi Winthrop Inc.). Additional blood samples were collected at 24, 48, 72, and 96 h.

### 2.3. Blood Collection and Sample Processing

All blood samples were obtained by intracardiac venipuncture under manual restraint. A 1-mL syringe fitted with a 25-gauge needle was used and pre-treated with lithium heparin.

Approximately 0.3 mL blood was collected at each sampling point and transferred into Eppendorf tubes. Blood samples were centrifuged immediately after collection at 5000× *g* for 10 min using a mini-centrifuge (MyFuge^®^ mini-spin, Benchmark Scientific C1008-G-E, Edison, NJ, USA). Plasma UA concentrations were analyzed within 20 min after centrifugation using a wet chemistry analyzer (Mindray BS-230, Mindray Biomedical Electronics, Nanchang, China). UA concentrations were determined using a uricase–peroxidase colorimetric assay (Clonatest UA, Linear Chemicals SLU, Barcelona, Spain).

### 2.4. Statistical Analysis

Statistical analyses were performed using MedCalc version 23.5.2 (64-bit). Descriptive statistical analyses of plasma UA concentrations included assessment of normality using the Kolmogorov–Smirnov test, as well as measures of central tendency and dispersion. These included minimum and maximum values, mean, 95% confidence interval (CI) for the mean, median, 95% CI for the median, standard deviation, relative standard deviation, and 5th and 95th percentiles.

Because the data were not normally distributed, non-parametric methods were used. The Kruskal–Wallis test was applied to compare plasma UA concentrations among different time points and treatment protocols. Serial measurement analysis was used to calculate and compare summary measures between groups, including minimum and maximum values, time to reach minimum and maximum values, and area under the curve. Area under the curve (AUC) was calculated for each individual animal using the Serial measurement analysis function in MedCalc. AUC was calculated over the sampling period using zero as the baseline value, consistent with the trapezoidal approach commonly used for serial concentration–time data.

### 2.5. Ethical Approval

The study protocol was reviewed and approved by the Animal Experimentation Ethics Committee of the Complutense University of Madrid under approval number 29/2025.

## 3. Results

All animals exhibited normal feeding behaviour and activity throughout the experimental period. No changes in behaviour, food intake, weight, defecation, or any clinical signs attributable to the experimental procedures were observed.

According to the Kolmogorov–Smirnov test, FNR, CR, and MR showed non-normal distribution (*p* < 0.05, Kolmogorov–Smirnov test), whereas FR, CNR, and MNR did not show significant deviation from normality (*p* < 0.05, Kolmogorov–Smirnov test). The obtained data for plasma UA concentrations under the six different experimental conditions are summarized in Table 1.

### 3.1. Fasting Without Rasburicase (Fnr) and Fasting with Rasburicase (Fr)

**Figure 1:**
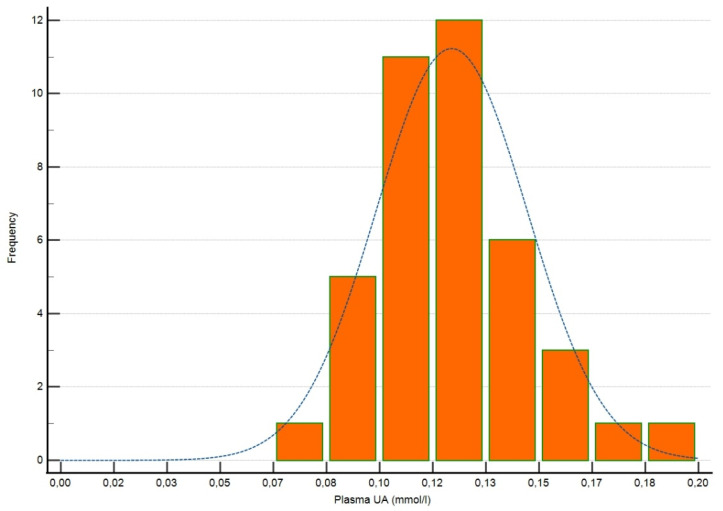
Histogram of plasma UA concentrations in fasting snakes without rasburicase administration (FNR group).

**Figure 2:**
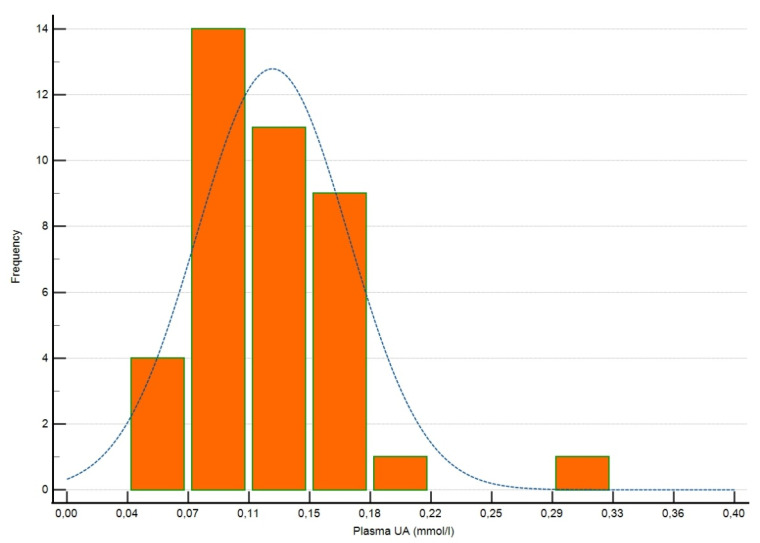
Histogram of plasma UA concentrations in fasting snakes with rasburicase administration (FR group).

Plasma UA concentrations under fasting followed a non-normal distribution in untreated snakes (FNR, Kruskal–Wallis test, *p* = 0.0115) and normal distribution in snakes treated with rasburicase (FR, Kruskal–Wallis test, *p* = 0.2288). The data distribution in both groups is depicted in Figure 1 and Figure 2.

**Figure 3:**
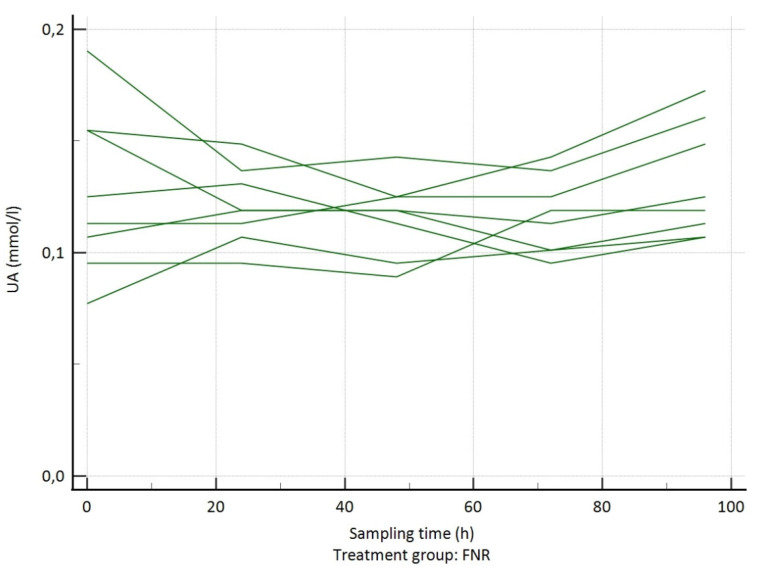
Evolution of plasma UA concentrations at different time points in fasting snakes without rasburicase administration (FNR group). The lines represent the trajectory of UA concentrations for each individual animal.

**Figure 4:**
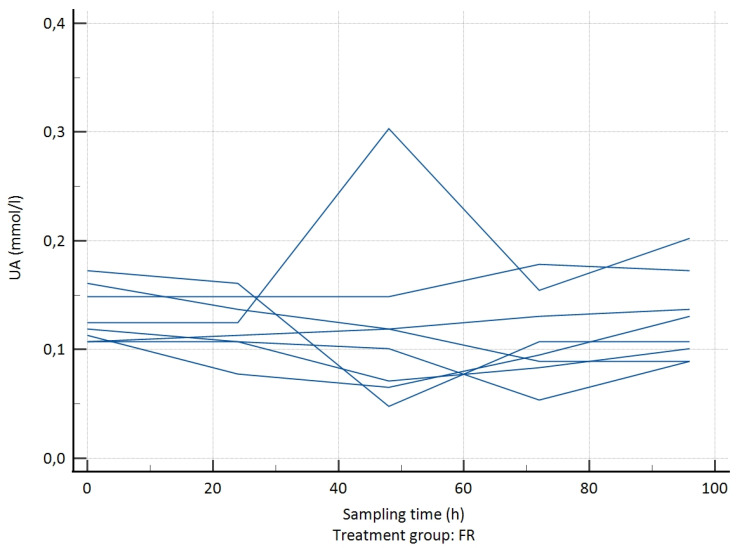
Evolution of plasma UA concentrations at different time points in fasting snakes receiving rasburicase (0.2 mg/kg IM) (FR group). The lines represent the trajectory of UA concentrations for each individual animal.

Rasburicase administration produced a mean reduction in plasma UA concentration of 7.2% in the first 24 h. However, no statistically significant differences in plasma UA concentrations were detected among individuals at any of the sampling times according to the Kruskal–Wallis test in either group (FNR: *p* = 0.8264; FR: *p* = 0.6135) (Figure 3 and Figure 4). Likewise, no significant differences were found between the FNR and FR groups overall (*p* = 0.6295).

### 3.2. Consumption of Thawed Day-Old Chicks Without (Cnr) and with Rasburicase (Cr) Administration

**Figure 5:**
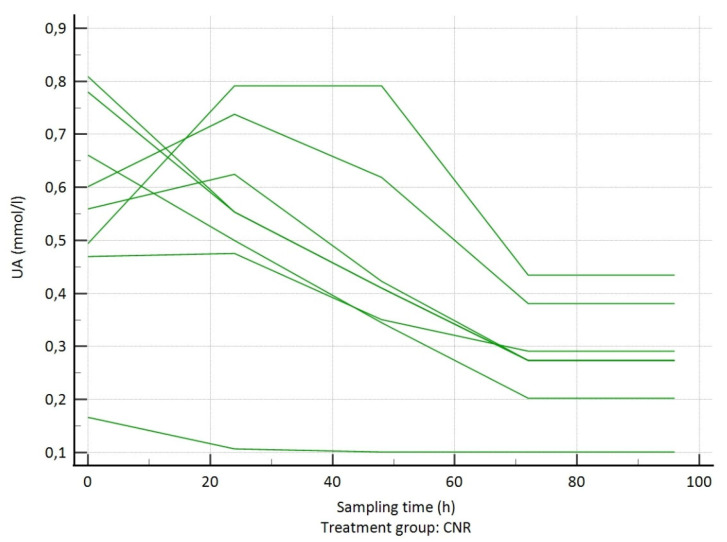
Evolution of plasma UA concentrations at different time points following administration of day-old chicks without rasburicase administration (CNR group). The lines represent the trajectory of UA concentrations for each individual animal.

**Figure 6:**
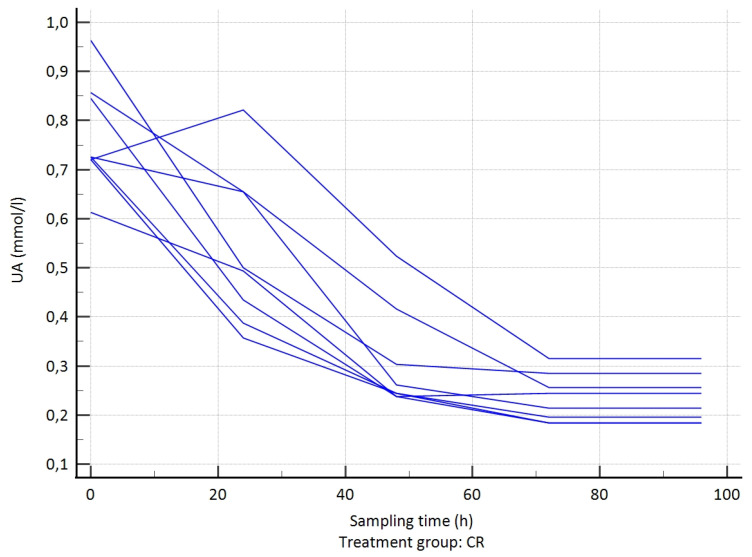
Evolution of plasma UA concentrations at different time points following administration of day-old chicks and rasburicase (0.2 mg/kg IM) (CR group). The lines represent the trajectory of UA concentrations for each individual animal.

After the ingestion of the thawed chick, plasma UA concentrations increased by a median of 30% (range 21–60%) within 48 h following ingestion of day-old chicks. In some individuals, peak concentrations were observed at 48 h rather than at 24 h (Figure 5 and Figure 6).

Rasburicase administration resulted in mean reductions in plasma UA concentrations of 29% at 24 h and 59% at 48 h compared with time 0 values, corresponding to mean decreases of 0.23 ± 0.09 mmol/L and 0.45 ± 0.11 mmol/L, respectively. UA concentrations appeared to stabilize near baseline by 72 h after time 0, corresponding to day 4 after feeding, with a more pronounced decline observed in the rasburicase-treated group (Figure 6).

No statistically significant differences were found between the group that did not receive rasburicase (CNR) and the group treated with rasburicase (CR) following chick ingestion (*p* = 0.907). However, significant differences were observed between different sampling times in both groups (CNR: Kruskal–Wallis test, *p* = 0.0029, and CR: Kruskal–Wallis, *p* = 0.000007).

Serial measurement analysis showed no differences between CNR and CR groups in minimum value, time to reach minimum value, or area under curve (*p* > 0.05), but the CR group showed a significantly higher maximum difference versus the first UA value (*p* = 0.0148).

### 3.3. Consumption of Thawed Mouse Without (Mnr) and with Rasburicase (Mr) Administration

**Figure 7:**
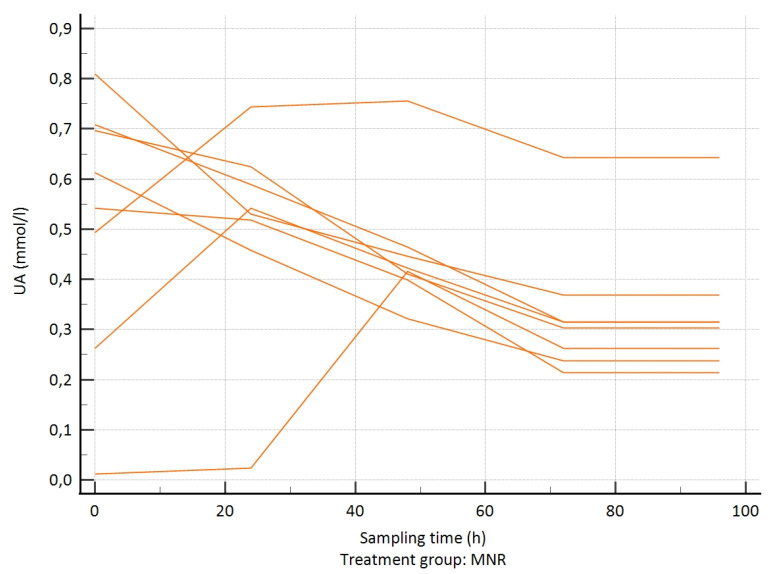
Evolution of plasma UA concentrations at different time points following a thawed mouse ingestion (MNR group). The lines represent the trajectory of UA concentrations for each individual animal.

**Figure 8:**
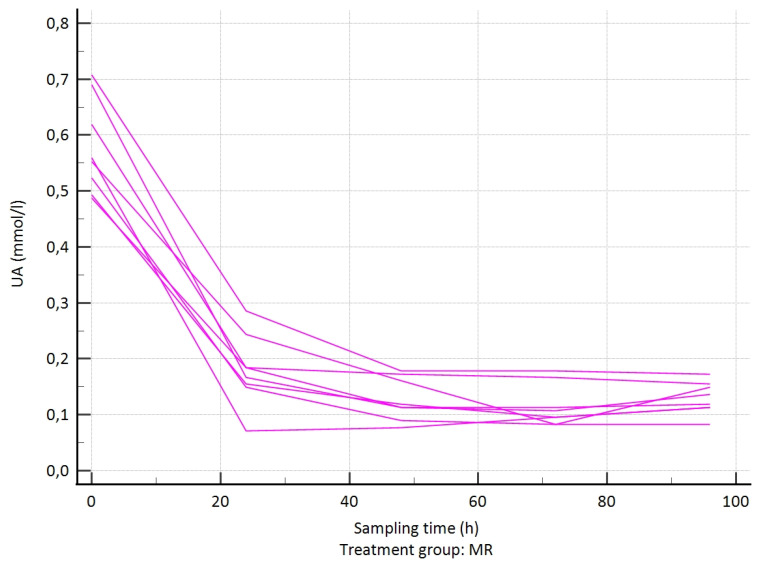
Evolution of plasma UA concentrations at different time points following a thawed mouse ingestion and rasburicase administration (0.2 mg/kg IM) (MR group). The lines represent the trajectory of UA concentrations for each individual animal.

Ingestion of the thawed mouse produced plasma UA concentration increases of 69–78% across time points (median 71%). Peak UA concentrations occurred between 24 and 48 h after feeding (Figure 7 and Figure 8).

A significant difference was detected between the group fed mice without rasburicase (MNR) and the group fed mice with rasburicase administration (MR) (Mann–Whitney U test on pooled data; *p* < 0.001). Significant differences were also observed among sampling times within both groups (MNR: Kruskal–Wallis test, *p* = 0.00326; MR: Kruskal–Wallis test, *p* = 0.0001).

Serial measurement analysis showed no significant difference between the MNR and MR groups in the time to reach the minimum value (*p* > 0.05). However, the MR group showed a significantly lower minimum value (*p* = 0.0054), a greater maximum difference from the first UA value (*p* = 0.0002), and a lower area under the curve (*p* = 0.0003).

Rasburicase administration resulted in mean reductions in plasma UA concentrations of 69% at 24 h and 78% at 48 h compared with time 0 values, corresponding to mean decreases of 0.36 ± 0.11 mmol/L and 0.38 ± 0.09 mmol/L, respectively (Figure 8). Plasma UA concentrations appeared to stabilize near baseline by day 4 after feeding, corresponding to 72 h after time 0, in the untreated group (MNR), and within 48 h in the rasburicase-treated group (MR) (Figure 7 and Figure 8).

**Figure 9:**
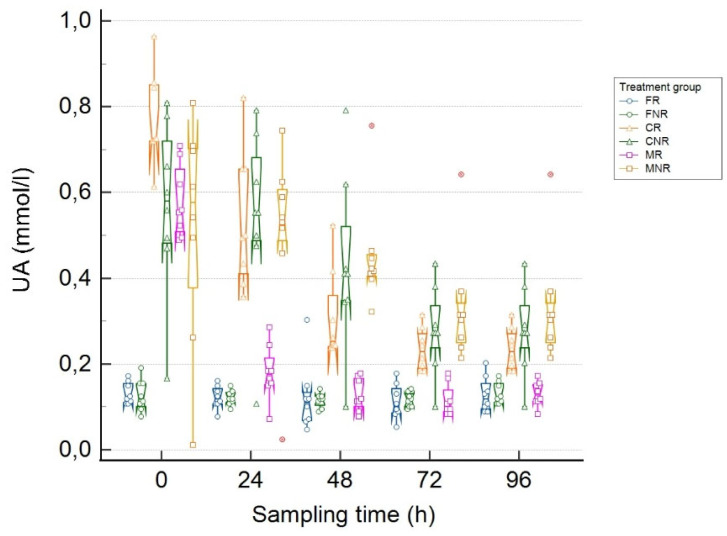
Box-and-whisker plot representing plasma UA concentrations at different time points in the different experimental treatments. FNR: Fasting No Rasburicase; FR: Fasting Rasburicase; CNR: Chick No Rasburicase; CR: Chick Rasburicase; MNR: Mouse No Rasburicase; MR: Mouse Rasburicase.

A graphic representation of UA concentrations in all six different groups is provided in Figure 9.

## 4. Discussion

To the authors’ knowledge, this is the first study evaluating the duration of action and efficacy of a single intramuscular dose of rasburicase in reducing plasma UA concentrations in ball pythons (*Python regius*) under different physiological conditions.

Throughout the six experimental phases, all snakes consistently accepted food whenever it was offered, despite repeated handling and the prolonged study period. One phase of the study was conducted during winter; however, all animals continued to feed normally. This was most likely related to the maintenance of stable environmental temperatures and photoperiods throughout the experimental period, as well as to the regular feeding schedule. The absence of seasonal anorexia under controlled husbandry conditions should therefore be interpreted as an expected response rather than as an unexpected finding. Although seasonal reductions in activity and food intake may occur in some snakes, *Python regius* does not undergo true brumation in the wild, but rather a period of reduced activity during the dry season, more consistent with aestivation than with full torpor. Temperature reductions of approximately 5 °C below the optimal maintenance range of 27–32 °C have been reported to induce brumation-like physiological responses in pythons [23,24]. In the present study, such environmental changes were not implemented, which likely contributed to the maintenance of normal feeding behavior throughout the study.

Analysis of the results identified one individual in the chick without rasburicase group (CNR) that did not show an increase in plasma UA concentrations after feeding (Figure 5). This may have contributed to the lack of statistically significant differences between the CNR and CR groups, with significant differences being detected only among sampling time points. No additional analysis was performed after excluding the data from the snake that showed no variation in plasma UA concentrations. A possible explanation is an unrecorded husbandry-related event, in which the animal may have failed to consume the day-old chick and the prey item was subsequently removed during routine enclosure maintenance.

In the mouse without rasburicase group (MNR), three individuals reached peak plasma UA concentrations between 48 and 72 h after mouse ingestion (Figure 7). Previous studies have shown that plasma UA concentrations may peak between one and four days after consumption of a mouse meal [5]. Therefore, the interindividual variability observed in the present study is consistent with the expected physiological response in these animals, as UA kinetics in snakes are known to vary depending on maintenance temperature and the amount of food digested [23].

**Figure 10:**
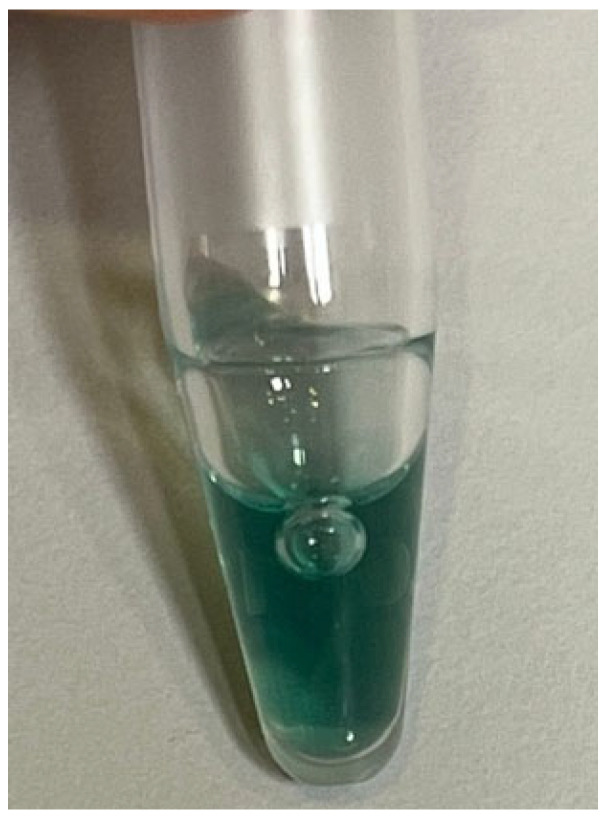
Green plasma observed in one ball python included in the study.

Another notable individual variation involved the presence of green-colored plasma (Figure 10) in one snake following mouse ingestion during both the MNR and MR experiments. Because UA concentrations were determined using a colorimetric assay, plasma pigmentation may have interfered with the analytical results obtained for this individual [24,25]. Elevated bilirubin concentrations and hemolysis have previously been reported to affect UA measurements obtained using colorimetric methods [5]. Likewise, biliverdin, which is responsible for the green coloration of plasma, may also have influenced the measurements. Although most reptiles possess colorless or slightly yellow plasma, green plasma has previously been reported in iguanas, arboreal lizards, and snakes [26,27]. Future studies should consider the analysis of paired samples using the uricase–peroxidase colorimetric assay in parallel with HPLC or a non-peroxidase-coupled analytical method, in order to further evaluate potential methodological differences in plasma UA measurement.

In all experimental groups receiving rasburicase, plasma UA concentrations decreased, although the magnitude of the reduction varied among treatments. In fasting animals, no apparent decrease in UA concentrations was observed following rasburicase administration, and concentrations remained relatively stable throughout the five-day monitoring period. This finding may reflect the fact that baseline UA concentrations were already low, providing insufficient substrate for the enzymatic activity of rasburicase. Alternatively, the pharmacological effect of the drug may simply be less detectable at low UA concentrations. In contrast, studies performed in freshwater turtles of the genus *Trachemys*, which exhibit lower baseline UA concentrations, demonstrated a measurable reduction following rasburicase administration [21]. Another factor that should be considered is the potential for ex vivo UA degradation by circulating rasburicase between sample collection and analysis [19], which could theoretically mask a small in vivo effect. However, in the present study, samples were centrifuged immediately after collection and analyzed without delay, making this effect unlikely to have had a major impact on the results. This consideration may be more relevant in clinical settings in which blood samples are submitted to external laboratories, as delayed processing could allow continued ex vivo enzymatic degradation of UA and result in falsely decreased measured UA concentrations. Therefore, prompt sample processing should be considered when interpreting UA concentrations in animals treated with rasburicase.

In the chick-feeding experiments, rasburicase administration resulted in a greater reduction in plasma UA concentrations than that observed in untreated animals, with mean decreases of 29% at 24 h and 59% at 48 h, corresponding to 0.23 ± 0.09 mmol/L and 0.45 ± 0.11 mmol/L, respectively. By day 5 after feeding, rasburicase-treated snakes showed lower plasma UA concentrations, suggesting a faster return toward baseline values.

The effect was even more pronounced in snakes fed mice. In this group, rasburicase administration resulted in mean reductions in plasma UA concentrations of 69% at 24 h and 78% at 48 h compared with time 0 values, corresponding to mean decreases of 0.36 ± 0.11 mmol/L and 0.38 ± 0.09 mmol/L, respectively. As observed in the chick-fed animals, plasma UA concentrations returned toward baseline earlier in rasburicase-treated snakes than in untreated controls.

These findings differ from those reported in one of the few available studies evaluating the efficacy of rasburicase in reptiles, which included five red-eared sliders (*Trachemys scripta*) and five Greek tortoises (*Testudo graeca*). Although that study was presented as an abstract at an international conference and has not been published in a peer-reviewed journal, rasburicase was reported to reduce UA concentrations by approximately 70%, with effects persisting for up to 21 days [21]. Comparison of these findings with those of the present study suggests that rasburicase may exert a stronger and longer-lasting effect on Chelonians than in snakes. However, alternative explanations should also be considered. Previous studies have shown that increased protein intake in uricotelic species leads to increased UA production [23], which may provide greater substrate availability for rasburicase activity. Conversely, lower endogenous UA clearance in Chelonians could also contribute to a more prolonged apparent drug effect. Further studies directly comparing reptile taxa under standardized conditions are required to clarify these potential differences.

No adverse effects attributable to rasburicase administration were observed throughout the study. All animals maintained normal behavior, appetite, and defecation patterns. In human medicine, adverse reactions have been described, particularly with older non-recombinant uricase formulations, which were associated with higher rates of hypersensitivity due to impurities in their preparation [17].

Repeated administration of rasburicase in humans has been associated with the development of anti-rasburicase antibodies; however, these antibodies are generally non-neutralizing and typically appear between one and six weeks after treatment [17]. In the present study, there was no evidence suggestive of antibody development. The mouse with rasburicase group (MR), which was evaluated last, showed the greatest reduction in plasma UA concentrations, with values 40–60% lower than those observed in the untreated mouse-fed group and 30–40% lower than those observed in the rasburicase-treated chick-fed group. This finding does not support a clinically relevant reduction in rasburicase efficacy over the course of the study. Similarly, no signs consistent with hypersensitivity reactions were observed [17,20]. Nevertheless, because the experimental design followed a fixed sequence rather than a randomized crossover design, a period effect cannot be formally excluded. This should be considered when interpreting the apparent absence of immunogenicity or loss of efficacy after repeated administration.

Hematocrit, hemoglobin concentration, and the presence of methemoglobin were not evaluated, and the only assessment supporting the absence of hemolysis was the observation of normal urate coloration in the treated snakes. In addition, the nucleated erythrocytes of reptiles may be less susceptible to rasburicase-induced hemolysis. Therefore, future studies should investigate the occurrence of hemolysis more thoroughly, and statements indicating that rasburicase does not cause hemolysis should be made with caution.

One limitation of this study is the relatively small sample size of eight animals, which reduced statistical power and limited the ability to detect subtle differences among treatment groups. In addition, interindividual variability may have had a greater influence on the overall results because of the limited number of animals included. Exploratory analysis of pooled time-point data suggested differences between groups; however, these results were interpreted with caution because repeated measurements from the same animals are not independent. Therefore, greater emphasis was placed on animal-level summary measures derived from serial measurement analysis.

Another limitation is that the pharmacokinetics of rasburicase were not evaluated in this species. The present study only assessed its pharmacodynamic effect on plasma UA concentrations. Future studies should investigate the pharmacokinetic profile of rasburicase in reptiles or directly measure circulating rasburicase activity, as has been performed in human medicine [19].

Another potential avenue for future research would be to evaluate the combined use of rasburicase, which promotes UA degradation, and allopurinol, which inhibits UA synthesis. However, studies in human oncology patients have reported comparable outcomes between rasburicase monotherapy and combined rasburicase–allopurinol therapy [28].

Finally, regarding drug safety, simultaneous measurement of plasma allantoin and UA concentrations would have been valuable to confirm the metabolic conversion of UA by rasburicase. Allantoin is considerably more water-soluble than xanthine, the metabolite that accumulates following inhibition of UA synthesis by allopurinol [11], and is therefore presumed to be less toxic when present at increased concentrations. Increased plasma xanthine concentrations have been associated with renal failure in carnivorous avian species treated with allopurinol [9]. Direct measurement of allantoin would have helped confirm in vivo enzymatic conversion and exclude the alternative explanation of ex vivo UA degradation within the sample tube.

## 5. Conclusions

The present study demonstrates that a single intramuscular administration of rasburicase at a dose of 0.2 mg/kg is well tolerated in clinically healthy ball pythons (*Python regius*), with no observable adverse effects during the study period. Rasburicase did not significantly affect baseline plasma UA concentrations in fasting animals. However, following food intake, particularly after ingestion of mice, the drug significantly reduced plasma UA concentrations and accelerated their return to baseline values. These findings indicate that the apparent efficacy of rasburicase, as measured by plasma UA reduction, is influenced by substrate availability and becomes more evident during periods of increased UA production. Although the study was conducted in healthy animals experiencing physiological postprandial hyperuricemia rather than pathological hyperuricemia, the results suggest that rasburicase may represent a promising therapeutic option for reducing elevated UA concentrations in reptiles. Its rapid mechanism of action and favorable safety profile support further investigation of its clinical utility. Future studies should focus on the pharmacokinetics of rasburicase in reptiles, evaluate its long-term safety, and determine its efficacy in clinical cases of hyperuricemia, gout, and renal disease. In addition, investigations involving larger sample sizes and different reptile taxa would help to establish evidence-based treatment protocols for the management of urate-related disorders in reptile medicine. Clinical use in hyperuricaemic or gouty patients cannot yet be recommended based on this study alone, since extrapolation from physiological postprandial hyperuricaemia to pathological hyperuricaemia carries non-trivial assumptions about substrate availability, enzyme saturation, and concurrent renal dysfunction.

## Figures and Tables

**Table 1:** Summary statistics of plasma uric acid (UA) concentrations in ball python under the six experimental treatments.

	Plasma UA (mmol/L)					
Treatment Group	FNR	FR	CNR	CR	MNR	MR
**N**	40	40	40	40	40	40
**Minimum**	0.0774	0.0476	0.101	0.184	0.0119	0.0714
**Maximum**	0.190	0.303	0.809	0.964	0.809	0.708
**Mean**	0.123	0.123	0.420	0.418	0.428	0.227
**95% confidence interval for the mean**	0.115 to 0.130	0.109 to 0.138	0.354 to 0.485	0.343 to 0.492	0.367 to 0.489	0.167 to 0.286
**Median**	0.119	0.116	0.411	0.309	0.414	0.155
**95% confidence interval for the median**	0.113 to 0.125	0.107 to 0.131	0.292 to 0.488	0.248 to 0.474	0.315 to 0.510	0.115 to 0.176
**Standard Deviation**	0.02368	0.04536	0.2050	0.2330	0.1914	0.1874
**Relative Standard Deviation**	0.1932	0.3683	0.4882	0.5580	0.4473	0.8270
**5–95 Percentiles**	0.0922 to 0.167	0.0595 to 0.190	0.101 to 0.791	0.184 to 0.851	0.119 to 0.750	0.0803 to 0.655
**Kolmogorov–Smirnov normality test, *p* value**	0.0115 *	0.2288	0.2535	<0.0001 *	0.2320	<0.0001 *

FNR: fasting without rasburicase; FR: fasting with rasburicase; CNR: chick without rasburicase; CR: chick with rasburicase; MNR: mouse without rasburicase; MR: mouse with rasburicase. * Non-normal distribution according to the Kolmogorov–Smirnov test (*p* < 0.05).

## Data Availability

The data presented in this study are available on request from the corresponding author. The data are not publicly available due to privacy restrictions.
